# Gefitinib in patients with progressive high-grade gliomas: a multicentre phase II study by Gruppo Italiano Cooperativo di Neuro-Oncologia (GICNO)

**DOI:** 10.1038/sj.bjc.6603669

**Published:** 2007-03-13

**Authors:** E Franceschi, G Cavallo, S Lonardi, E Magrini, A Tosoni, D Grosso, L Scopece, V Blatt, B Urbini, A Pession, G Tallini, L Crinò, A A Brandes

**Affiliations:** 1 Department of Medical Oncology, Bellaria Hospital, Bologna, Italy; 2 Department of Medical Oncology, Istituto Oncologico Veneto – IRCCS, Padova, Italy; 3 Department of Pathology, Bellaria Hospital, Bologna, Italy; 4 Department of Medical Oncology, S Anna Hospital, Ferrara, Italy

**Keywords:** high-grade gliomas, gefitinib, EGFR, Akt

## Abstract

To investigate the role of gefitinib in patients with high-grade gliomas (HGGs), a phase II trial (1839IL/0116) was conducted in patients with disease recurrence following surgery plus radiotherapy and first-line chemotherapy. Adult patients with histologically confirmed recurrent HGGs following surgery, radiotherapy and first-line chemotherapy, were considered eligible. Patients were treated with gefitinib (250 mgday^−1^) continuously until disease progression. The primary end point was progression-free survival at 6 months progression-free survival at 6 months (PFS-6). Tissue biomarkers (epidermal growth factor receptor (EGFR) gene status and expression, phosphorylated Akt (p-Akt) expression) were assessed. Twenty-eight patients (median age, 55 years; median ECOG performance status, 1) were enrolled; all were evaluable for drug activity and safety. Sixteen patients had glioblastoma, three patients had anaplastic oligodendrogliomas and nine patients had anaplastic astrocytoma. Five patients (17.9%, 95% CI 6.1–36.9%) showed disease stabilisation. The overall median time to progression was 8.4 (range 2–104+) weeks and PFS-6 was 14.3% (95% CI 4.0–32.7%). The median overall survival was 24.6 weeks (range 4–104+). No grade 3–4 gefitinib-related toxicity was found. Gefitinib showed limited activity in patients affected by HGGs. Epidermal growth factor receptor expression or gene status, and p-Akt expression do not seem to predict activity of this drug.

Despite recent progress in the treatment of high-grade gliomas (HGG), survival rates remain dismal. Genetic signatures of chemoresistance, such as epidermal growth factor receptor (EGFR) amplification and overexpression and activation of phosphatidyl-inositol 3-kinase (PI3K)/Akt pathway, are present in a consistent proportion of patients with HGG (Kleihues and WK, 2000).

At time of recurrence after surgery and radiotherapy, with or without adjuvant chemotherapy, only a few options are available: repeat surgery, re-irradiation in selected cases and/or chemotherapy. However, responses with the currently available chemotherapy are unsatisfactory. At the time of second recurrence after first-line chemotherapy, hardly any of the options available are worth pursuing. Even in anaplastic oligodendroglial tumours, that are historically considered chemosensitive tumours, second-line treatments seem to be scarcely effective (Triebels *et al*, 2004).

In this setting, novel agents such as new chemotherapy compounds and anticancer agents against specific molecular targets, have therefore been investigated. The protein, EGFR, one of the most widely studied treatment targets, is a tyrosine kinase receptor of the erbB family that is commonly implicated in several different human tumour types. After ligand binding (EGF, TFG-alpha, amphiregulin) EGFR undergo activation and, by recruiting adaptor or signalling proteins, cause proliferative and antiapoptotic stimuli to spread into the cell. Epidermal growth factor receptor-activated pathways include the mitogen-activated protein kinase and the Akt cascades important for cell proliferation and survival, respectively.

Small molecule tyrosine kinase inhibitors (TKIs), such as gefitinib and erlotinib, interact with the intracellular domain of EGFR by interfering with the intermolecular phosphorylations of key tyrosine kinases in the activation loop of catalytic TK domains, subsequently blocking the EGFR signalling pathways (Mendelsohn and Baselga, 2003). Epidermal growth factor receptor is an intriguing target in HGG because it is usually overexpressed and the EGFR gene is amplified in most cases (Kleihues and WK, 2000).

Gefitinib (IRESSA) acts as an ATPmimetic, binding the intracytoplasmic ATP pocket domain and blocking receptor phosphorylations and EGFR-mediated downstream pathways. Preclinical studies have shown the potential activity of gefitinib in intracranial tumours (Heimberger *et al*, 2002). Recently, in a phase II trial on recurrent glioblastoma (GBM) by Rich *et al* (2004b) gefitinib administration at a dose of 500 mgday^−1^ achieved a stable disease rate of 42% and a median event-free survival of 8.1 weeks.

In the present multicentre phase II trial of the Gruppo Italiano Cooperativo di Neuro-Oncologia (GICNO), the activity and safety profile of oral gefitinib at the dose of 250 mgday^−1^ was evaluated in patients with recurrent/progressive HGG, who had undergone surgery, radiotherapy and chemotherapy.

An analysis was made of EGFR protein expression, gene status and the PI3K/Akt pathway activation status by using the phosphorylated Akt protein (p-Akt) expression.

## PATIENTS AND METHODS

### Treatment plan

Gefitinib was administered orally at a dose of 250 mgday^−1^ until disease progression (PD) and/or significant clinical decline, unacceptable toxicity or the patient decision to withdraw. Toxicity was graded using the National Cancer Institute Common Toxicity Criteria, version 2.0 (NCI-CTC v2.0).

For grade 2 skin rashes and diarrhoea not tolerated by the patient, Gefitinib was suspended until the symptoms resolved. In patients with other significant grade 2 nonhematologic toxicities, treatment was withheld until the condition/symptoms resolved; in those with grade 3 or 4 toxicity, treatment was discontinued, and the patient was re-evaluated until toxicity was grade ⩽1. Patients with unresolved toxicity after 2 weeks were withdrawn from the study.

### Patient selection

Eligibility criteria included: age ⩾18 years; life expectancy >8 weeks; histological diagnosis of progressive HGG (GBM, anaplastic astrocytoma, anaplastic oligodendroglioma and anaplastic oligoastrocytoma) according to the WHO 2000 classification. Other eligibility criteria were: ECOG performance status ⩽2; stable corticosteroid dose for at least 2 weeks before enrolment; normal laboratory values for hepatic, renal and bone marrow function. Patients on enzyme-inducing antiepileptic drugs (EIAEDs) were considered eligible. Stable corticosteroids doses were mandatory because of the effect on p450 cytochrome (Vecht *et al*, 2003), and the compliance to response evaluation criteria in neuro–oncology (Macdonald *et al*, 1990).

All the patients had undergone contrast computed tomography or magnetic resonance imaging of the brain showing at least one contrast-enhancing measurable lesion (⩾1 cm), indicating PD or recurrence after surgery, radiotherapy and no more than one line of chemotherapy. None of the patients had undergone chemotherapy in the 4 weeks before entering the study (6 weeks for patients that received BCNU chemotherapy). All patients gave their fully informed consent in writing to take part in the study, which was approved by the Institutional Review Board (Bellaria Hospital, Bologna, Italy), and was conducted according to the principles of the Declaration of Helsinki and the rules of Good Clinical Practice.

### Statistical analysis

In this multicentre, open-label, single-arm, phase II trial (1839IL/0116) we evaluated the activity and toxicity profile of gefitinib (IRESSA) 250 mgday^−1^ in HGG patients and any correlation with molecular biomarkers. The primary endpoint was progression-free survival at 6 months (PFS-6). This single-stage study of 28 assessable patients was designed to differentiate between a 6-month PFS rate of 15 and 35% with *α*=0.05 and *β*=0.8.

Patients' clinical and radiological evaluation was performed every 8 weeks, or sooner if clinically indicated, according to Macdonald's criteria (Macdonald *et al*, 1990). Any variation in the neurologic status and in the daily dosage of corticosteroids were recorded.

The secondary end points were safety, time to progression (TTP) and overall survival (OS). Tissue biomarkers (EGFR gene status and expression, (p)Akt expression) were also evaluated.

Differences between and among groups were compared using Fisher's exact test or Pearson's *χ*^2^ test for the following qualitative variables: age (<60 or ⩾60), gender (male *vs* female), histological grade (WHO grade 3 *vs* 4 tumours), ECOG PS (0–1 *vs* 2), use of EIAEDs (yes *vs* no), acneiform skin rash (presence *vs* absence), diarrhoea (presence *vs* absence), EGFR and p-Akt protein expressions (positive *vs* negative) and EGFR gene status (genetic gain (amplified+polysomic) *vs* diploid). Time to progression and OS were calculated using the Kaplan–Meier method; different groups were compared using the log-rank test. All statistical tests were two sided, and statistical significance was defined as *P*<0.05. All analyses were performed using the statistical package SPSS version 11.0 (SPSS Italia Srl, Bologna, Italy).

#### Tissue preparation

Tumour specimens, obtained before administration of cancer therapy were embedded in paraffin. Gene copy number per cell was investigated by FISH using the LSI EGFR SpectrumOrange/CEP7 SpectrumGreen probe (Vysis, Abbott Laboratories, IL, USA), according to a published protocol (Hirsch *et al*, 2003). Patients were classified according to the frequency of tumour cells with specific numbers of copies of the EGFR gene and chromosome 7 centromere: (1) disomy (two copies in >90% of cells); (2) polysomy (⩾4 copies in ⩾10% of cells); (3) gene amplification (presence of a tight EGFR gene cluster and an EGFR gene to chromosome ratio of ⩾2 copies per cell in ⩾10% of cells).

Epidermal growth factor receptor protein expression was evaluated by immunohistochemistry (IHC) with the mouse antihuman EGFR, clone 31G7 monoclonal antibody (Zymed Laboratories, San Francisco, CA, USA) using methods and assessment criteria described elsewhere (Hirsch *et al*, 2003).

Using the rabbit antimouse P-Akt (Ser 473) polyclonal antibody (Cell Signaling Technology, Beverly, MA, USA), according to the manufacturer's instructions. Phosphorylated Akt and EGFR expression were scored based on intensity and fraction of positive cells as described elsewhere. (Cappuzzo *et al*, 2005). Tumour samples were considered positive for p-Akt in patients with nuclear staining intensity of at least 1+ in >10% of tumour cells. The total score for EGFR protein expression was calculated by multiplying the membrane staining intensity score and the fraction score producing a total range of 0–400. For statistical analyses, scores of 0–300 were considered negative/low expression, and scores of 301–400 were considered positive/high expression.

Positive controls were chosen on non-small-cell lung cancer specimens verified in previous studies from our group, in which we found a statistical correlation between increased EGFR protein expression and increased gene copy number.

All the negative controls were incubated with nonimmune solution in place of primary antibody.

## RESULTS

Twenty-eight Caucasian patients were enrolled between February and December 2003. Their pretreatment characteristics, including age, histology, performance status, surgery and dose of radiotherapy, chemotherapy and antiepileptic treatments are summarised in Table 1. All patients were evaluable for response.

### DP and response

Progression-free survival at 6 months and PFS-12 were 14.3%. (95%CI 4.0–32.7%) and 7.1% (95%CI 0.9–23.5%), respectively.

**Figure 1:**
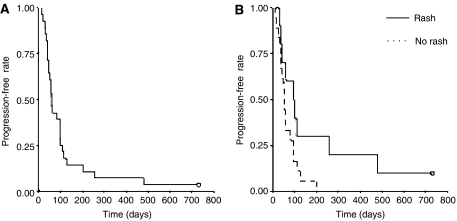
Kaplan–Meier curves for progression-free survival. (**A**) In overall population; (**B**) by rash.

The median TTP was 8.4 weeks (range 4–104+). Progression-free survival at 6 months and PFS-12 were both 12.5% (95%CI 1.6–38.4%) in the GBM subgroup (Figure 1A and B).

The overall disease-control rate was 17.9% (95%CI 6.1–36.9%) with 0 PR and 5 s.d. (three GBM, one anaplastic astrocytoma and one anaplastic oligodendroglioma), in the GBM subgroup was 12.5% (95%CI 1.6–38.4%) with 2 s.d. Three of four patients with disease control at 6 months were receiving EIAEDs. No differences between patients that achieved s.d. against those progressing were recorded by histology, age, corticosteroids and EIAEDs' use.

Time to progression was significantly correlated with rash (*P*=0.05) (Figure 1B) but not with gender, histological tumour grade, ECOG performance status, and use of EIAEDs and/or age.

### OS

The median OS was 24.6 weeks (range 4–104+), OS at 6 months was 50% (95%CI 30.7–69.4%) and OS at 12 months, 14.3% (95%CI 4.0–32.7%).

Overall survival was statistically correlated with disease control (*P*=0.005) but not with gender, histological tumour grade, ECOG performance status, use of EIAEDs, age and the molecular biomarkers evaluated.

### Toxicity

Grade 3/4 adverse events occurring during gefitinib treatment were: grade 3 diarrhoea (*n*=1) and neutropenia (*n*=1), grade 4 acute pulmonary oedema (*n*=1), pulmonary thromboembolism (*n*=1) and central nervous system haemorrhage (*n*=1). The investigators did not find any temporal and/or causal correlation between the gefitinib treatment and these adverse events. Grade 1/2 gefitinib-related acneiform skin rash was common (32.1%).

### Molecular biomarkers

**Figure 2:**
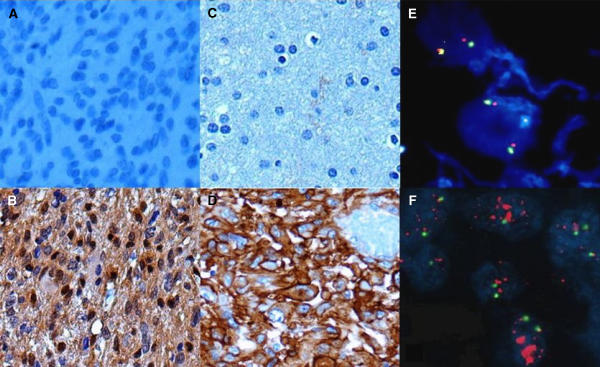
P-Akt IHC expression examples: (**A**) negative, (**B**) positive; EGFR IHC expression examples: (**C**) negative, (**D**) positive, EGFR gene status by FISH examples: (**E**) diploid, (**F**) amplified.

The analysis of EGFR expression, p-Akt expression and EGFR genetic status was performed on tissue samples from 21 patients; results were available in 21, 21 and 19 cases, respectively (Figure 2). Seven patients did not give informed consent for biomarkers analysis. Epidermal growth factor receptor protein overexpression was significantly correlated with EGFR genetic gain (*P*=0.01) but not with p-Akt expression. No correlations were found between molecular biomarkers and PFS-6 (Table 2).

## DISCUSSION

In a recent phase II trial, Rich *et al* (2004b) evaluated the role of gefitinib at a dose of 500 mgday^−1^ in 57 patients with recurrent GBM. None of the patients presented objective responses, and a PFS-6 of 13.2% was achieved. Patients on EIAEDs received a gefitinib dose escalation to 750–1000 mgday^−1^, and the authors concluded that gefitinib was active in GBM patients. Epidermal growth factor receptor protein expression and gene status and EGFRvIII protein expression were not significantly correlated with PFS-6 and OS.

In the present trial, the activity and toxicity profile of gefitinib at a dose of 250 mgday^−1^ were evaluated in patients with HGG. This dose was chosen following the experience of gefitinib use in lung cancer treatment. In this setting, two large, randomised phase II trials, investigated effectiveness and toxicity of gefitinib at 250 mg or 500 mgday^−1^. No difference was found between response rates and survivals following the two different dose schedules, whereas the adverse event rates were higher in 500 mgday^−1^ arms (Fukuoka *et al*, 2003; Kris *et al*, 2003). Moreover, data generated from phase I studies of gefitinib showed that responses and disease stabilizations also occur at doses lower than 250 mgday^−1^ and gefitinib has been shown to be effective at a dose of 50 mgday^−1^ in intermittent schedules (Nakagawa *et al*, 2003). Also, PFS-6 does not appear to be related to the dose escalation of a similar orally active TK inhibitor, erlotinib in GBM patients on EIAEDs (Cloughesy *et al*, 2005).

We found an overall PFS-6 of 14.3%, and a PFS-6 of 12.5% in the GBM subgroup. Disease control was achieved in five patients, and three of the four patients with disease control at 6 months were on EIAEDs.

However, the PFS-6 rate was too low to consider the drug active in HGG.

Interestingly, the disease control obtained with gefitinib seemed to provide HGG patients with a clinical benefit. A possible explanation for these results may be that some glioma cells are sensitive to gefitinib, even at low doses.

Molecular signatures of EGFR-TKI sensitivity have recently been found in NSCLC: in particular, specific point mutations in the ATP pocket domain correlate with dramatic responses to gefitinib (Lynch *et al*, 2004; Paez *et al*, 2004; Pao *et al*, 2004). Overall, however, these mutations do not fully explain the whole spectrum of gefitinib activity: responses and disease stabilisations have been described in wild-type EGFR patients.

Furthermore, these mutations in the EGFR tyrosine kinase domain have not been found in patients with glioma (Barber *et al*, 2004; Rich *et al*, 2004a; Marie *et al*, 2005). Rich *et al* (2004a) showed that gefitinib-treated GBM patients with an event-free survival of more than 24 weeks did not harbour such EGFR point mutations . New indicators of EGFR TKIs sensitivity in patients without EGFR point mutations are therefore required.

Findings in preclinical and clinical studies have highlighted the activation status of the Akt protein (Cappuzzo *et al*, 2004; Sordella *et al*, 2004).Akt, a serine/threonine kinase acts downstream of EGFR to regulate many cellular processes, including cell survival, proliferation and growth, and is activated by phosphorylation.

In the present study, we assessed EGFR protein expression, gene amplification status and P-Akt protein expression, in tumours without EGFR tyrosine kinase point mutations, such as HGG, in order to understand why disease is controlled, sometimes for a long-lasting period, by gefitinib in a fraction of patients.

However, we did not find any significant correlation between disease control and molecular biomarkes.

Recently, other molecular biomarkers have been evaluate in the analyses of two groups. Haas-Kogan *et al* (2005) showed that response to erlotinib was associated with EGFR expression and amplification. These correlations were stronger and statistically significant among the 29 patients with GBM (*P*=0.03 and 0.02, respectively). Among six responders with sufficient tumour tissue, none presented the EGFR truncated version known as EGFRvIII. Low levels of p-Akt expression was also associated with TTP (*P*<0.001).

Another study conducted retrospectively by Mellinghoff *et al* (2005) on patients with GBM treated with two different EGFR TKIs (gefinitib and erlotinib) showed results at least partially in contrast with previous cited studies. In fact responses to gefitinib were recorded for the first time and the importance of EGFRvIII expression (especially in presence of PTEN expression) on EGFR TKIs activity assessed in gliomas. However, in this study unusual response evaluation criteria were used (patients were considered responders if a <25% of tumour reduction was achieved , clinical conditions and corticosteroid dosages were not considered).

Despite a suggestion of a predictive role of PI3K pathway inactivation by the latter two studies, no convincing molecular predictor of EGFR TKIs efficacy have emerged.

Even in our analysis EGFR expression or amplification and p-Akt expression, do not seem to provide useful information to select HGG patients that could have a benefit from gefitinib.

The optimal biological dosage of gefitinib has yet to be defined. Nor is it known whether gefitinib has a place in the treatment of patients with HGG. However, further investigations should be conducted to establish whether molecular determinants of clinical effect can be used for patient selection in the treatment of GBM.

## Figures and Tables

**Table 1:** Patients' pretreatment characteristics

Characteristic (*n*=28)	*n* (%)
	
*Gender*	
Male	17 (61)
Female	11 (39)
	
*Age*	
Median	55
Range	29–70
	
*ECOG performance status*	
0	3 (11)
1	21 (75)
2	4 (14)
	
*Histology*	
GBM	16 (57)
Anaplastic oligodendroglioma	3 (11)
Anaplastic astrocytoma	9 (32)
	
*Previous treatments*	
Surgery/biopsy	28 (100)
Radiotherapy	27 (96)
Chemotherapy	26 (93)
	
*Antiepileptic drugs*	
None	4 (14)
EIAEDs	21 (75)
Non-EIAEDs	3 (11)

ECOG=Eastern Cooperative Oncology Group; EIAEDs=enzyme-inducing antiepileptic drugs; GBM=glioblastoma.

**Table 2:** Molecular biomarkers analysis and correlations with PFS-6

	**N (%)**	**PFS-6**	**P**
*EGFR by FISH (n=19)*			
Genetic gain	9 (47.3%)	0	NS
Disomy	10 (52.7%)	30%	
			
*EGFR by IHC (n=21)*			
Positive	8 (38.1%)	0	NS
Negative	13 (61.9%)	23%	
			
*p-Akt by IHC (n=21)*			
Positive	10 (47.6%)	10%	NS
Negative	11 (52.4%)	18%	

EGFR=Epidermal growth factor receptor; FISH=fluorescence *in situ* hybridization; IHC=immunohistochemistry; NS, nonsignificant; p-Akt=phosphorylated Akt; PFS-6=progression-free survival at 6 months.
